# Long-term follow-up in a Chinese child with congenital lipoid adrenal hyperplasia due to a *StAR* gene mutation

**DOI:** 10.1186/s12902-018-0307-6

**Published:** 2018-11-06

**Authors:** Xiu Zhao, Zhe Su, Xia Liu, Jianming Song, Yungen Gan, Pengqiang Wen, Shoulin Li, Li Wang, Lili Pan

**Affiliations:** 1 Department of Endocrinology, Shenzhen Children’s Hospital, 7019# Yitian Road, Futian District, Shenzhen, 518038 Guangdong Province China; 2 Pathology Department, Shenzhen Children’s Hospital, Shenzhen, 518038 China; 3 Radiology Department, Shenzhen Children’s Hospital, Shenzhen, 518038 China; 4 Pediatrics Research Institute, Shenzhen Children’s Hospital, Shenzhen, 518038 China; 5 Department of Urology, Shenzhen Children’s Hospital, Shenzhen, 518038 China

**Keywords:** Congenital lipoid adrenal hyperplasia, Steroidogenic acute regulatory protein, Mutation, Growth

## Abstract

**Background:**

Congenital lipoid adrenal hyperplasia (CLAH) is an extremely rare and the most severe form of congenital adrenal hyperplasia. Typical features include disorder of sex development, early-onset adrenal crisis and enlarged adrenal glands with fatty accumulation.

**Case presentation:**

We report a case of CLAH caused by mutations in the steroidogenic acute regulatory protein (*StAR*) gene. The patient had typical early-onset adrenal crisis at 2 months of age. She had normal-appearing female genitalia and a karyotype of 46, XY. The serum cortisol and adrenal steroids levels were always nearly undetectable, but the adrenocorticotropic hormone levels were extremely high. Genetic analysis revealed compound heterozygous mutations at c. 229C > T (p.Q77X) in exon 3 and c. 722C > T (p.Q258X) in exon 7 of the *StAR* gene. The former mutation was previously detected in only two other Chinese CLAH patients. Both mutations cause truncation of the StAR protein. The case reported here appears to be a classic example of CLAH with very small adrenal glands and is the second reported CLAH case with small adrenal glands thus far. In a 15-year follow-up, the patient’s height was approximately average for females before age 4 and fell to − 1 SDS at 10 years of age. Her bone age was similar to her chronological age from age 4 to age 15 years.

**Conclusions:**

In conclusion, this is a classic case of CLAH with exceptionally small adrenal glands. Q77X mutation seems to be more common in Chinese CLAH patients. Additionally, this is the first report of the growth pattern associated with CLAH after a 15-year follow-up.

## Background

Congenital lipoid adrenal hyperplasia (CLAH, OMIM 201710) is the most severe form of congenital adrenal hyperplasia (CAH) and may cause 46, XY disorders of sex development (DSD). CLAH is an autosomal recessive inherited disorder caused by mutations of the steroidogenic acute regulatory protein (*StAR*; OMIM 600617) gene [1]. Currently, more than 83 different mutations of the *StAR* gene have been reported in approximately 190 patients [2]. However, only 17 cases of CLAH have been reported in Chinese patients [3–9]. The clinical features include severe adrenal and gonadal steroidogenesis defects due to disorder of the conversion of cholesterol to pregnenolone (P). All affected individuals with classic CLAH are phenotypically female regardless of their gonadal sex [1]. Hormone replacement enables long-term survival for these patients, but the literature regarding their long-term outcomes is limited.

Here, we report a 15-year follow-up of a Chinese patient with typical clinical manifestations of CLAH except for small adrenal glands.

## Case presentation

**Fig. 1:**
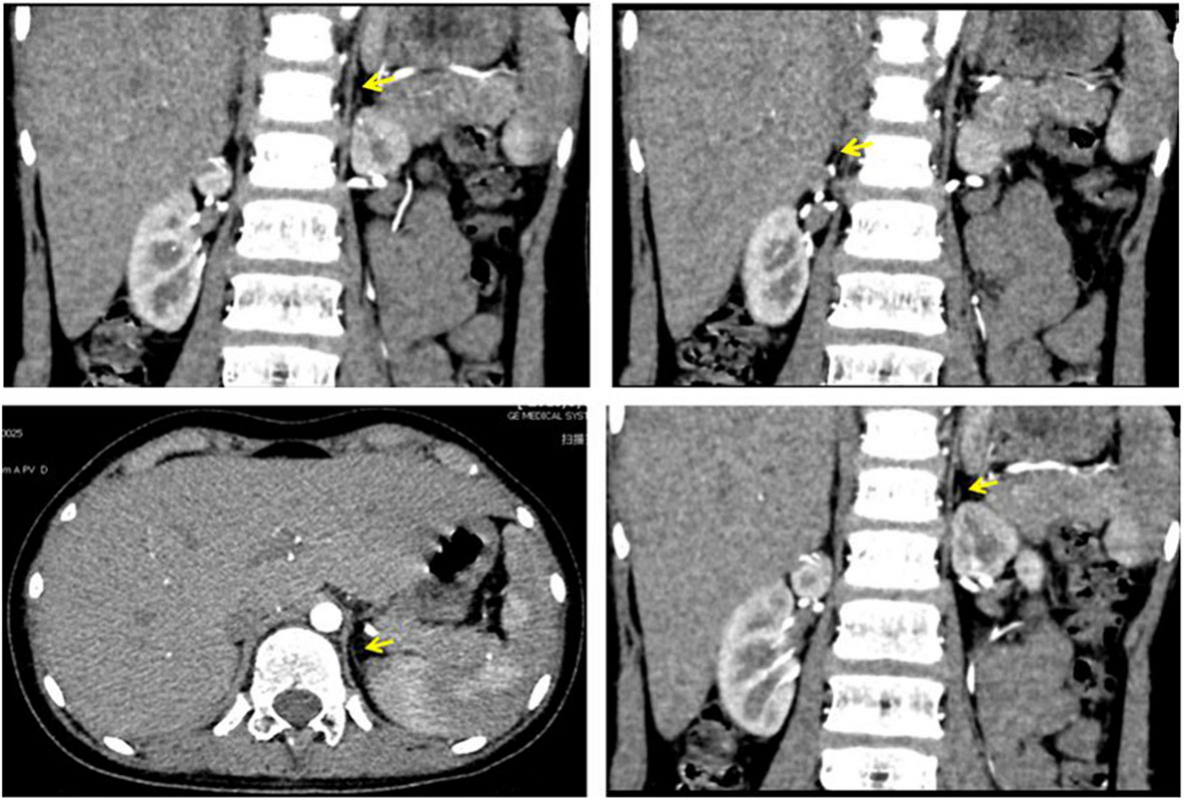
At the age of 13.2-year adrenal computed tomography (CT) scans revealed that the adrenal glands on both sides were much smaller than normal. The yellow arrows indicate the adrenal gland

**Fig. 2:**
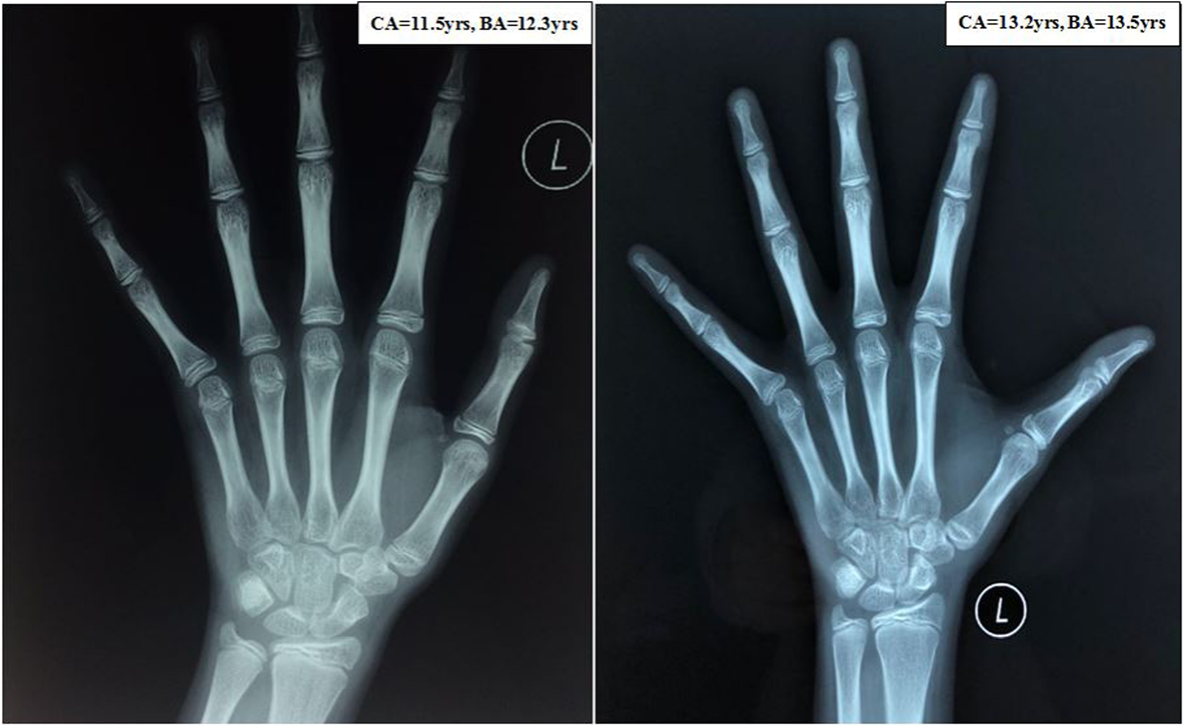
Bone age of the patient. *CA* chronological age, *BA* bone age, yrs. = years old

The patient was Chinese individual who was raised as a female. She was born full-term by vaginal delivery. She was the fourth child of nonconsanguineous parents. She was admitted to our hospital at 2 months of age with recurrent vomiting, diarrhea and dehydration. She was found to have hyperpigmentation, hyponatremia (sodium 114 mmol/L) and hyperkalemia (potassium 6.98 mmol/L). The serum adrenocorticotropic hormone (ACTH) level was greater than 1250 pg/ml accompanied by very low levels of cortisol (0.17 μg/dl) and aldosterone (4.21 pg/ml). The levels of all adrenal steroids, including testosterone (T), P, dehydroepiandrosterone sulfate, androstenedione and 17-hydroxyprogesterone, were always nearly undetectable. She was diagnosed with primary adrenal insufficiency and was prescribed hydrocortisone and 9ɑ-fludrocortisone orally [10]. However, her treatment compliance was poor. She often did not take the medications on time or at the recommended dose. She was followed up irregularly. Several episodes of adrenal crisis occurred, and she was admitted again at the age of 13.2 years. On admission, her blood pressure was 90/60 mmHg, and her height, weight and body mass index (BMI) were 148.7 cm (− 1.3 SDS), 35.3 kg (− 1.5 SDS) and 15.96 kg/m^2^ (− 1.0 SDS), respectively. Hyperpigmentation was found on her tongue, gums, face, trunk, elbows, knees, palms, and soles. The patient was in Tanner stage 1. External female genitalia were observed, including a normal-sized clitoris and a normal urethral orifice and vaginal orifice. No palpable gonads were identified in the inguinal or labial regions. No pubic or axillary hair was observed. She had a high ACTH level and lower levels of cortisol, adrenal steroids and aldosterone. She exhibited gonadal dysgenesis, including: 1) high baseline levels of luteinizing hormone (38.2 mIU/ml) and follicle-stimulating hormone (68.3 mIU/ml); 2) very low levels of baseline T (< 0.35 nmol/L) and T after administration of the human chorionic gonadotropin stimulation test (0.47 nmol/L); and 3) low levels of anti-Müllerian hormone and Inhibin b. The patient’s karyotype was 46, XY (big Y). Ultrasonography revealed the presence of testes-like masses in the pelvic cavity (1.4 cm × 0.8 cm × 1.0 cm on the left and 1.5 cm × 0.6 cm × 1.0 cm on the right). Neither ovaries nor a uterus was identified. After 13 years of steroid replacement therapy, the patient underwent an adrenal computed tomography (CT) scan at the age of 13.2 years. Adrenal CT scans revealed that the adrenal glands on both sides were much smaller than normal (Fig. 1). Magnetic resonance imaging of the abdominal and pelvic cavities showed no uterus or ovaries. The patient’s bone age (BA) was 13.5 years old (Fig. 2).

**Fig. 3:**
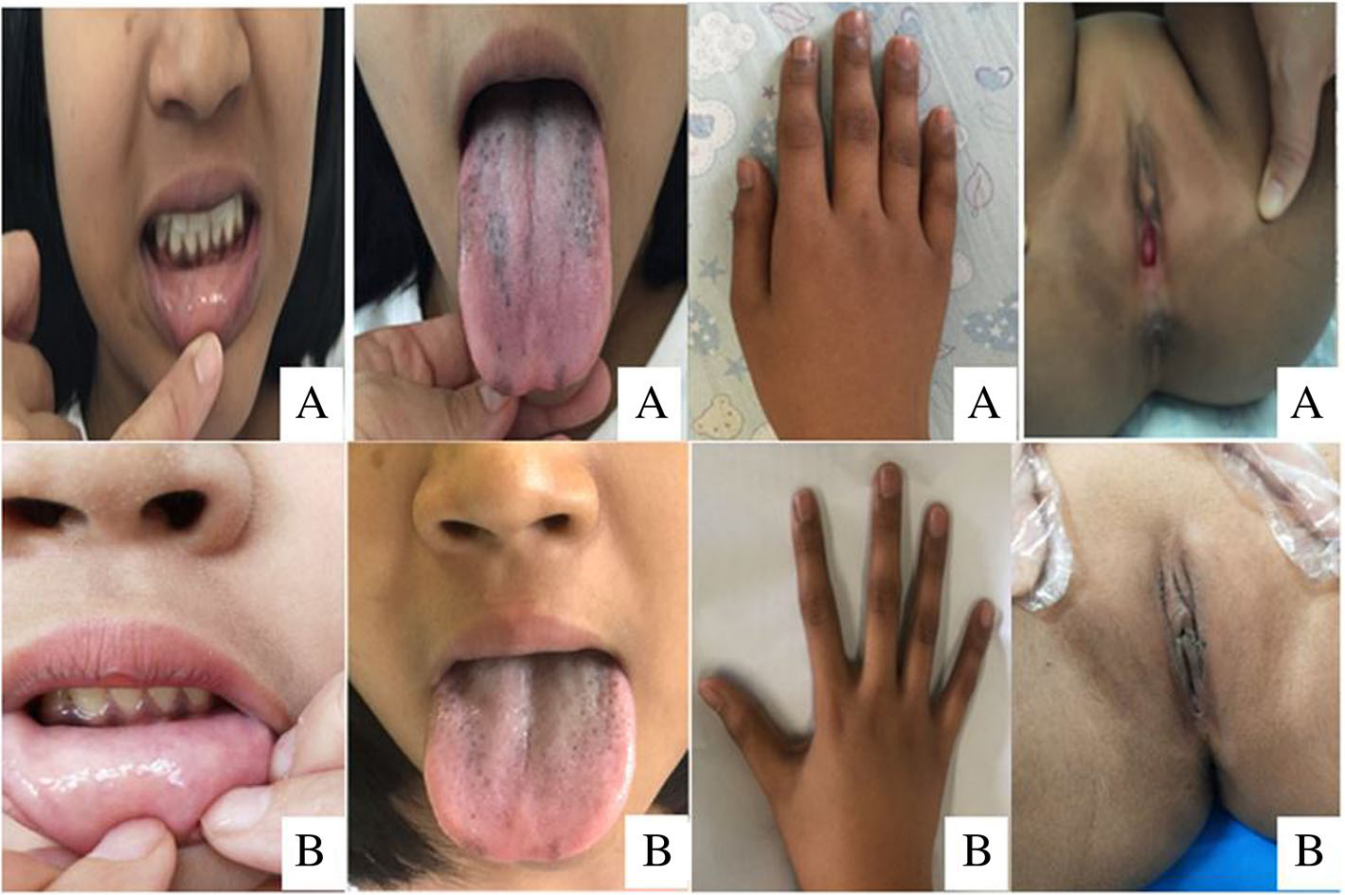
Photos of the patient with CLAH. (**a**) Photos at age 13.2 years, (**b**) Photos at age 15.5 years

**Fig. 4:**
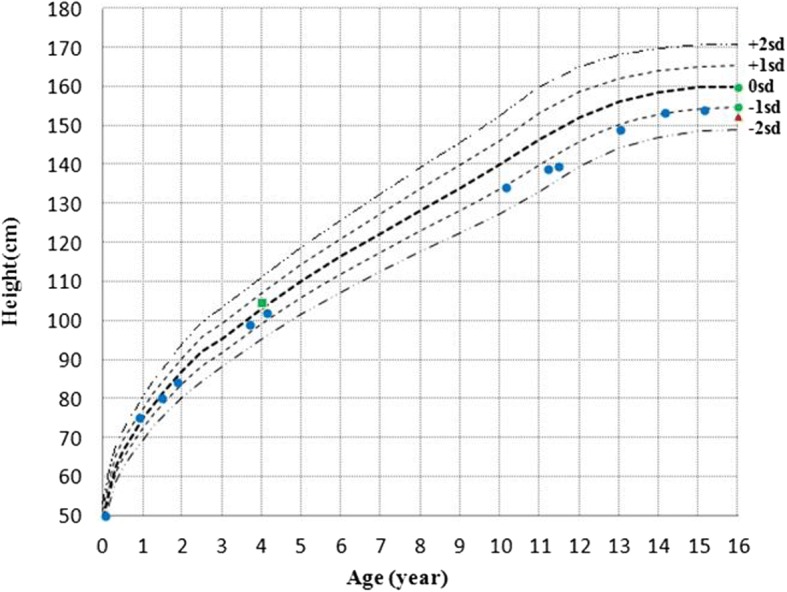
Chart showing the progression of the patient’s height during the 15-year follow-up. Blue dots indicate the patient’s height. Green dots indicate the height of the patient’s sisters. The red triangle represents the midparent height. The green square represents the height of the patient reported in Fu’s paper

The patient was given hydrocortisone (17.3 mg/m^2^ of body surface area per day) and 9ɑ-fludrocortisone (0.1 mg/day) regularly. Her hyperpigmentation was slightly alleviated, and she had no adrenal crisis episodes thereafter. At the age of 15.2 years, the patient began estrogen replacement therapy. After 4 months of estrogen treatment, her breasts began developing. Photos of the patient are shown in Fig. 3. Laboratory and treatment data were collected during the 15-year follow-up and are shown in Table 1. Growth data are shown in Table 2. Compared to healthy Chinese girls [11], the patient’s growth charts were drawn and are shown in Fig. 4. At 15.5 years old, she nearly reached her final adult height (154 cm), which was similar to her midparent height (152.5 cm) but shorter than the heights of her two sisters (156 cm, 160 cm).

**Table 1:** Laboratory test results and treatment of the patient during the 15-year follow-up

Age (year)	Variable	Cor	ACTH	17OHP	DHEAS	P	AD	LH	FSH	E2	T	PRL	Sodium	Chloride	Potassium	FBG	TC	TG	LDL	HDL	ATII	Renin	ALD	INH-b	AMH	HC	FC
	unit	μg/dl	pg/ml	ng/ml	μg/dl	ng/ml	ng/ml	mIU/ml	mIU/ml	pg/ml	nmol/L	ng/ml	mmol/L	mmol/L	mmol/L	mmol/L	mmol/L	mmol/L	mmol/L	mmol/L	pg/ml	ng/ml/hr	pg/ml	pg/ml	ng/ml	mg/m^2^/d	μg/d
	Normal range	4.5–23	0–53	0.16–1.85	45–139	0.1–8.45	0.3–1.97	0.3–15	0.6–25	20–85	0.35–2.1	5–35	135–145	96–105	3.5–5.5	3–5.6	3.1–5.18	0.23–1.7	2.07–3.37	0.9–1.04	23–75	0.15–2.33	10–160	92–147	5.6–18.8		
0.2		0.17	> 1250	0.1	0.11	1.4	< 0.3			< 20	< 0.35		114	82.2	6.98								4.21			18.7	100
0.3		0.51	1229		< 0.1						< 0.35		134.6	102.4	5.58											14.6	100
0.5		3.45	1229		< 0.1	0.57				< 20	< 0.35															17.2	100
0.9		0.92	> 1250		< 0.1	< 0.15				< 20	< 0.35															15.5	100
11.3		0.8	> 1250	0.03	0.1	< 0.15	< 0.3	40.8	58	< 20	< 0.35	16.4	128	93.2	5.2	4.5										17.5	100
13.2		0.67	723	0.04	0.8	< 0.15	< 0.3	38.2	68.3	< 20	< 0.35	21.15	130	97.6	4.79	4.7	5.39	1.03	3.54	0.85	48.97	1.73	114.87	79.28	4.03	17.3	100
14.2		0.11	804	0.07	0.9	0.3	< 0.3	35.1	49	< 20	< 0.35	22.71	138	103	4.4	4.5	5.51	1.25	3.68	0.81	57.91	0.5	113.4			16.2	100

*Cor* Cortisol, *ACTH* Adrenocorticotropic hormone, *P* Pregnenolone, *T* Testosterone, *DHEAS* Dehydroepiandrosterone sulfate, *AD* Androstenedione, *17OHP* 17-hydroxyprogesterone, *E2* Estradiol, *PRL* Prolactin, *FBG* Fasting blood glucose, *AT* Angiotensin, *ALD* Aldosterone, *TC* Cholesterol, *LDL* Low density lipoprotein, *HDL* High density lipoprotein, *TG* Triglyceride, *LH* Luteinizing hormone, *FSH* Follicle stimulating hormone, *INH-b* Inhibin b, *AMH* Anti-Müllerian hormone, *HC* Hydrocortisone, *FC* Fludrocortisone

**Table 2:** Physical assessments of the patient during the 15-year follow-up

Age (year)	BA (year)	Height (cm)	HtSDS	Weight (kg)	WtSDS	BMI (kg/m^2^)
Newborn		–		3.0	−0.6	–
0.2		50.0	0.2	3.7	1.1	14.80
0.9		72.5	−0.5	8.6	−0.1	15.27
1.5		80.0	−0.5	11.0	0.3	17.19
1.9		84.0	−0.1	12.0	0.5	17.01
3.7		99.0	−0.1	15.5	0.2	15.81
4.2	5	102.0	−0.3	18.0	0.9	17.30
10.2		134.0	−1.0	30.5	−0.2	16.99
11.3		138.8	−1.2	35.2	−0.1	18.27
11.5	12.3	139.5	−1.5	36.0	−0.4	18.50
13.2	13.5	148.7	−1.3	35.3	−1.5	15.96
14.2		153.3	−0.9	45.6	− 0.3	19.40
15.2	15	154.0	−1.1	52.4	0.3	22.09
15.5		154.0	−1.2	53.0	0.4	22.35

*HtSDS* Height standard deviation score, *WtSDS* Weight standard deviation score, *BMI* Body mass index, *BA* Bone age

The patient had no other diseases and exhibited normal intelligence. One of her elder sisters died during infancy from an unknown cause. Her parents and her other two sisters were healthy and underwent normal puberty. Her mother died in a traffic accident when she was 10 years old. No family history of DSD was noted.

Considering the above clinical picture, an initial diagnosis of 46, XY DSD caused by severe deficiency of adrenal and gonadal steroids was established. The most plausible causes of the patient’s condition include some specific types of CAH (3β-hydroxysteroid dehydrogenase deficiency, 17 hydroxysteroid dehydrogenase deficiency, CLAH) and congenital adrenal hypoplasia caused by mutations of the *NR0B1* or *NR5A1* genes [12]. Medical exome sequencing was the method of choice for a clear diagnosis.

### Gene analysis

**Fig. 5:**
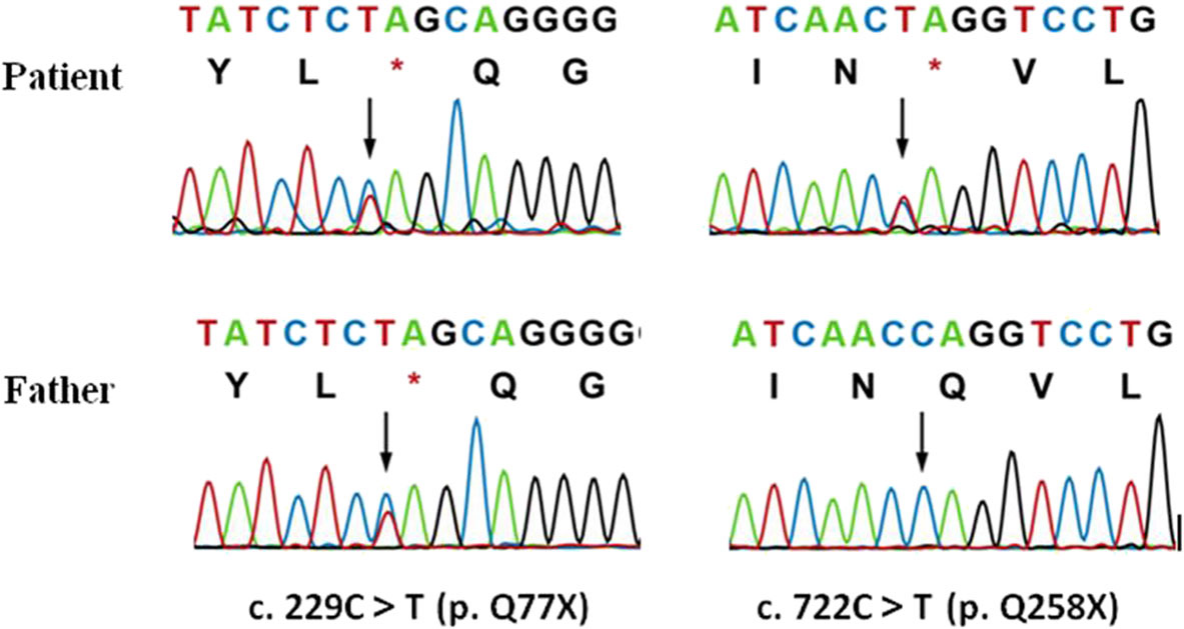
Sequence electropherograms showing the StAR gene mutations in the patient and her father. Sequence analysis of the StAR gene revealed two hemizygous nonsense mutations at c. 229C > T (p. Q77X) and c. 722C > T (p. Q258X). The heterozygous mutation of c. 229C > T (p. Q77X) was found in the patient’s father. Because the patient’s mother had died due to a traffic accident, the mother’s sequences could not be tested. The black arrows indicate the hemizygous nucleotides of c. 229C > T (p. Q77X) and c. 722C > T (p. Q258X) from the patient and the heterozygous mutation from the patient’s father

The sex-determining region on the Y chromosome gene was positive. Gene sequencing was performed with medical exome sequencing. The interpretation of the sequence variants and pathogenicity was conducted according to guidelines set by the American College of Medical Genetics and Genomics [13]. The Human Gene Mutation Database (HGMD), db SNP database, and 1000 database were used to identify whether the observed mutations have been reported previously. Compound heterozygous mutations were found in the *StAR* gene, with c. 229C > T (p. Q77X) in exon 3 and c. 722C > T (p. Q258X) in exon 7. The patient’s father had the same heterozygous mutation at c. 229C > T (p. Q77X) (Fig. 5). Unfortunately, the samples from her mother and her two sisters could not be obtained. No other variants were observed in the *CYP11A1*, *CYP17A1, HSD3β2, StAR, P450scc, MC2R, NR0B1* and *NR5A1* genes.

### Surgery and gonadal histology

**Fig. 6:**
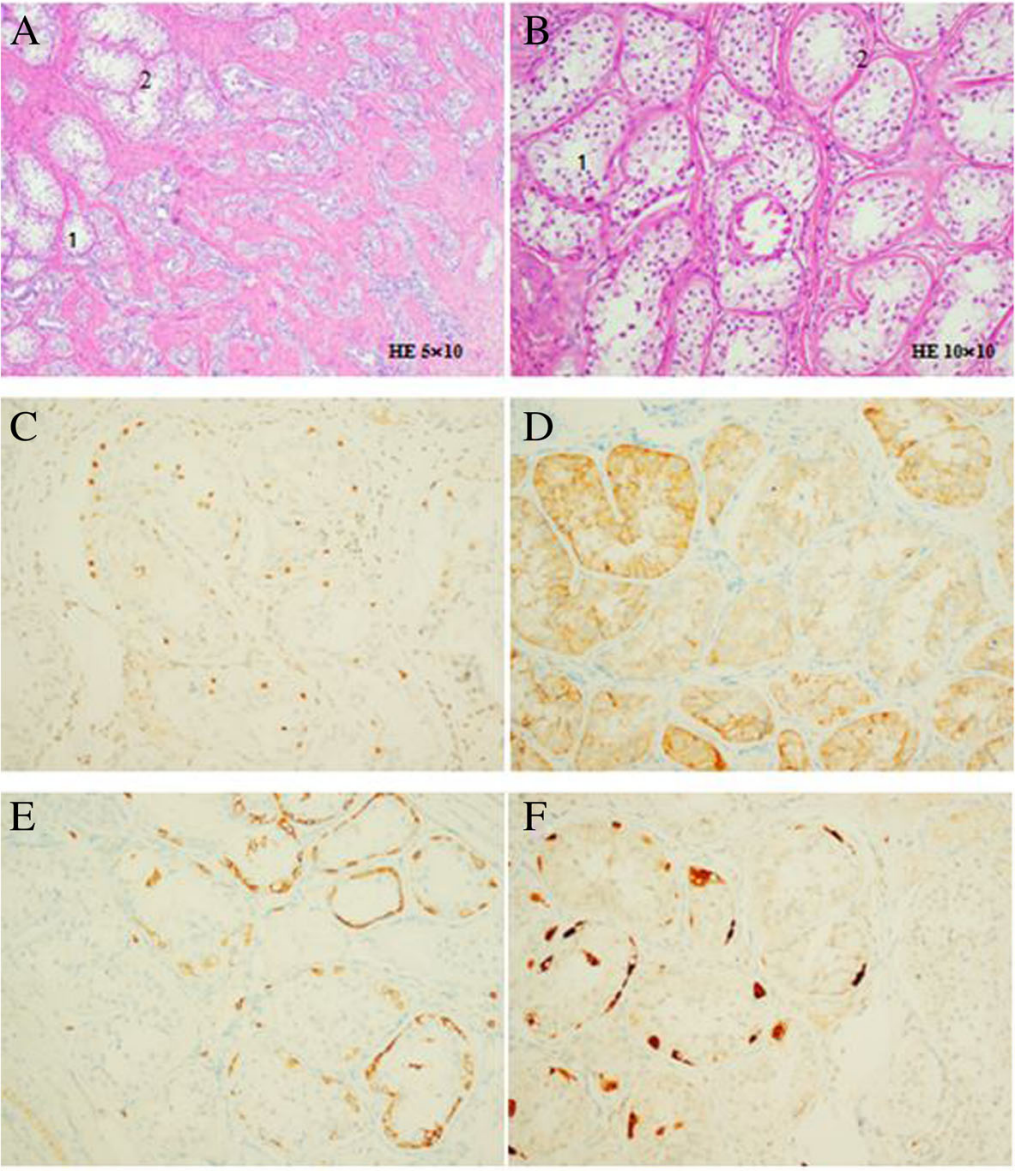
Histology of the removed gonads. **a** Testis at low magnification: Seminiferous tubules (1). Hyaline thickening of the basal membranes (2). **b** Testis at higher magnification: Seminiferous tubules (1). Hyaline thickening of the basal membranes (2). **c** Testis stained for OCT 3/4. **d** Testis stained for Inhibin A. **e** Testis stained for CD117. **f** Testis stained for TSPY1

After discussion with the DSD multidisciplinary team and consent from the patient and her father, the patient underwent surgery at 14.2 years old. Surgical exploration showed no Müllerian structures. Both gonads were removed, and the pathological results indicated hypogenesis of both testes. Hyalinization of the seminiferous tubules, the presence of spermatogonia and spermatocytes, and no sperm or sperm cells in the gonads were noted during the procedure. Immunohistochemical staining of the gonadal tissue was partly positive for CD117 and OCT 3/4 and positive for TSPY1 and Inhibin A (Fig. 6).

## Discussion and conclusion

CLAH is an autosomal recessive inherited disorder caused by to mutations in the *StAR* gene. The human *StAR* gene is located on chromosome 8p11.2 and consists of 7 exons that translate into a protein of 285 amino acids in length. *StAR* is required for translocation of cholesterol from the outer to the inner mitochondrial membranes to synthesize P [14] and is involved in the first step of adrenal and gonadal steroidogenesis. Individuals with mutations in this gene may have severe deficiency of adrenal and/or gonadal steroids accompanied by fatty accumulation in enlarged adrenal glands in most cases. The amino acid sequence of the StAR protein is highly conserved from 67 to 280 [4]. The full length of the protein consists of a mitochondrial target sequence on its N-terminus and a cholesterol-binding site on its C-terminus [15]. In vitro studies have revealed that the StAR protein without the C-terminal region had severe defects in function, while deletion of the N-terminus resulted in less loss-of-function [16].

We presented a case of CLAH caused by mutations in the *StAR* gene with a 15-year follow-up. This patient exhibited typical features of primary adrenal insufficiency shortly after birth. She was raised as a female and had visibly normal external female genitalia despite the presence of the male sex chromosome. The patient carried compound heterozygous mutations c. 229C > T (p. Q77X) and c. 722C > T (p. Q258X) in the *StAR* gene, both of which are nonsense mutations. Q258X is a known pathogenic hotspot mutation in Japanese, Korean and Chinese individuals with CLAH [5, 17]. Deleting 10 amino acids from the C-terminus has been shown to decrease StAR activity by approximately 50% [18]. With the nonsense mutation p. Q258X, StAR protein activity decreases to 16% of that of wild-type activity due to truncation of 27 amino acids in the C-terminal region [19].

**Fig. 7:**
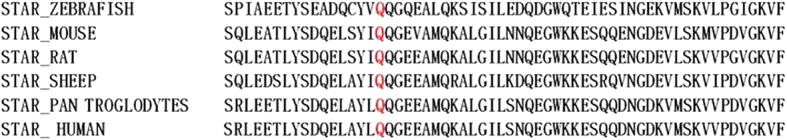
Sequence alignment of the StAR protein from seven species. Q77 is a highly conserved amino acid (highlighted in red)

The other mutation in our patient is c. 229C > T (p. Q77X) in exon 3. Q77 is a highly conserved residue from zebrafish (Fig. 7). The nonsense mutation c. 229C > T (p. Q77X) changes glutamine 77 to a terminal codon. Therefore, the StAR protein became truncated to a length much shorter than that of the Q258X mutant and may have exhibited less than 16% activity. Therefore, the Q77X mutation was predicted to be pathogenic. The Q77X mutation has been previously reported in only two other Chinese patients with CLAH [7, 9]. Therefore, this mutation may be more common in the Chinese population.

The typical features of CLAH are enlarged adrenal glands with lipid deposits [20]. However, a suspected diagnosis of CLAH in the absence of adrenal enlargement cannot be ruled out [21, 22]. Huang et al. [5] studied images of the adrenal glands from CLAH patients and found that 7 of 9 cases had enlarged adrenal glands, one had normal-sized adrenal glands with fatty deposits, and one had normal adrenal glands. Only one CLAH case with small-sized adrenal glands, as observed in our patient, has been reported previously [20] (Table 3); however, the paper provided no corresponding speculation. The physiological mechanism for small adrenal glands remains unclear.

**Table 3:** Data of two CLAH cases with small-size adrenals due to *StAR* gene mutation

Author	Ethnicity	Rearing gender	Consanguineous parents	Age at onset (month)	Manifestations	Adrenal imaging by CT	Treatment	Karyotype	Alleles	Mutation area	Gene mutation	Type of mutation
Bose et al. [20]	Guatemalan/Amerindian	Female	Yes	2	typical	The left adrenal gland was not seen, the right one was small	HC, FC	46, XY	H	Exon 6	c.InsA677	Frameshift
Our case	Chinese/Asian	Female	No	2	typical	Both were small	HC, FC	46, XY (big Y)	C	Exon 3 Exon 5	c. 229C>T c. 722C>T	Nonsense

*HC* Hydrocortisone, *FC* Fludrocortisone, *CT* Computed tomography, *H* Homozygote, *C* Compound heterozygote

Data regarding the growth patterns of CLAH patients are limited (Table 4). The heights of CLAH patients from 1.2 years to 4 years of age were reported to be between the 10th and 50th percentiles of that among the normal population [4, 23]. The final heights in patients with CLAH were reported to be shorter or similar to the calculated midparent height [9, 24, 25]. Here, we constructed the first growth curve of a CLAH patient before and after sex hormone replacement. Birth length and heights before age 4 were similar to the average value of the general population. However, her height decreased to approximately − 1 SDS at 10 years old. At 15.5 years old, the patient’s height was near her final adult height, which was similar to her midparent height but shorter than her two sisters’ heights. The patient’s BA was similar to her chronological age from age 4 to age 15.2 (Fig. 2), which is different from the retarded BA reported in other cases [24].

**Table 4:** Available growth data of CLAH cases due to *StAR* gene mutations

Author	Ethnicity	Relation	Age at onset (month)	Manifestations	Ht (percentile)	FAH (cm)	FAH vs MP	BA	Treatment	Karyotype	Alleles	Mutation
Khoury et al. [24]	French Canadian	siblings	11	typical	NM	NM	similar	significantly delayed	HC, FC, SH	46, XY	H	p.L275P
	French Canadian	siblings	1.5	typical	NM	NM	similar	slightly delayed	HC, FC, SH	46, XX	H	p.L275P
Fluck et al. [25]	Caucasian	siblings	10	typical	NM	143	low	NM	HC, FC	46, XX	C	p.T44HfsX3 p.G221S
	Caucasian	siblings	14	typical	NM	159.5	similar	NM	HC, FC	46, XY	C	p.T44HfsX3 p.G221S
Qiu et al. [9]	Chinese		1.3	typical	NM	152	low	NM	HC, FC, SH	46, XY	C	p.Q77X c.838delA
Fu et al. [4]	Chinese		11	typical	P50th (4 years)	NM	NM	NM	HC, FC	46, XY	H	p.K236Tfs∗47
Park et al. [23]	Korean	twins	1.3	typical	P3-10th (14 months)	NM	NM	NM	HC, FC	46, XX	C	p.R182C p.Q258X
	Korean	twins	< 1	typical	P10-25th (14 months)	NM	NM	NM	HC, FC	46, XX	C	p.R182C p.Q258X
Our case	Chinese		2	typical	P25-50th (18 months) P25th (4.2 years)	154	similar	NM	HC, FC, SH	46, XY	C	p.Q77X p.Q258X

*HC* Hydrocortisone, *FC* Fludrocortisone, *SH* Sex hormone, *CT* Computed tomography, *AI* Adrenal insufficiency, *GD* Gonadal dysplasia, *BA* Bone age, *NM* No mention, *H* Homozygote, *C* Compound heterozygote, *E* Estradiol

Limitations: Growth data in children with CLAH have rarely been analyzed. Our study plotted the patient’s anthological measurement data from infancy to adolescence. However, accurate descriptions of growth patterns in children with CLAH require more cases and longer follow-ups.

In conclusion, we identified a CLAH patient with a 15-year follow-up. Small-sized adrenal glands were the primary characteristic in our patient. Based on her long-term follow-up, her growth profile is the first reported growth pattern of CLAH. Our patient is the third reported case with the Q77X mutation in the *StAR* gene, which seems to be more common in the Chinese population.

## Abbreviations

ACTH: Adrenocorticotropic hormone
AHC: Adrenal hypoplasia, congenital
BA: Bone age
BMI: Body mass index
CLAH: Congenital lipoid adrenal hyperplasia
CT: Computed tomography
DSD: Disorders of sex development
P: Pregnenolone
SDS: Standard deviation score
SF-1: Steroidogenic factor-1
StAR: Steroidogenic acute regulatory protein
T: Testosterone
